# Hierarchical Alteration of Brain Structural and Functional Networks in Female Migraine Sufferers

**DOI:** 10.1371/journal.pone.0051250

**Published:** 2012-12-05

**Authors:** Jixin Liu, Ling Zhao, Guoying Li, Shiwei Xiong, Jiaofen Nan, Jing Li, Kai Yuan, Karen M. von Deneen, Fanrong Liang, Wei Qin, Jie Tian

**Affiliations:** 1 School of Life Sciences and Technology, Xidian University, Xi'an, China; 2 The 3rd Teaching Hospital, Chengdu University of Traditional Chinese Medicine, Chengdu, China; 3 Institute of Automation, Chinese Academy of Sciences, Beijing, China; University of Massachusetts Medical School, United States of America

## Abstract

**Background:**

Little is known about the changes of brain structural and functional connectivity networks underlying the pathophysiology in migraine. We aimed to investigate how the cortical network reorganization is altered by frequent cortical overstimulation associated with migraine.

**Methodology/Principal Findings:**

Gray matter volumes and resting-state functional magnetic resonance imaging signal correlations were employed to construct structural and functional networks between brain regions in 43 female patients with migraine (PM) and 43 gender-matched healthy controls (HC) by using graph theory-based approaches. Compared with the HC group, the patients showed abnormal global topology in both structural and functional networks, characterized by higher mean clustering coefficients without significant change in the shortest absolute path length, which indicated that the PM lost optimal topological organization in their cortical networks. Brain hubs related to pain-processing revealed abnormal nodal centrality in both structural and functional networks, including the precentral gyrus, orbital part of the inferior frontal gyrus, parahippocampal gyrus, anterior cingulate gyrus, thalamus, temporal pole of the middle temporal gyrus and the inferior parietal gyrus. Negative correlations were found between migraine duration and regions with abnormal centrality. Furthermore, the dysfunctional connections in patients' cortical networks formed into a connected component and three dysregulated modules were identified involving pain-related information processing and motion-processing visual networks.

**Conclusions:**

Our results may reflect brain alteration dynamics resulting from migraine and suggest that long-term and high-frequency headache attacks may cause both structural and functional connectivity network reorganization. The disrupted information exchange between brain areas in migraine may be reshaped into a hierarchical modular structure progressively.

## Introduction

Migraine is an idiopathic headache disorder that has been an important healthcare and social problem for its great influence on the quality of life, accompanied by severe headaches, nausea and light sensitivity [1]. Functional and structural neuroimaging has provided unique insights for evaluating brain alteration in migraine [2]. Several studies on structural differences in gray matter revealed that the brain altered its shape as a result of repetitive migraine attacks, and the structural differences in patients were associated with headache frequency [3], [4]. Functional connectivity analysis also found regional homogeneity abnormalities in the migraine-afflicted brain [5]–[7]. These studies focused on the local variation in migraine from different angles. Meanwhile, it has been suggested that properties of the underlying anatomical network may constrain the main organizing principles of functional connectivity [8], [9]; the functional connectivity, in turn, may reflect the underlying structural connectivity [10], [11]. Combining measures of altered functional and structural information from cortical networks may enrich our understanding of the mechanisms responsible for brain alteration during migraine [12]. So far, however, there has been little discussion on the coupling of brain functional and structural network dysregulation underlying the pathophysiology in migraine.

Due to frequent migraine-related nociceptive input, migraine has had serious secondary effects upon the central nervous system (CNS) [13], causing irregular functional connectivity in patients' cortical networks leading to irregular brain circuits associated with pain-related information processing [1]. Several studies demonstrated that chronic pain not only harmed brain regions involved in central pain processing but also resulted in selective alteration of cortical areas unrelated to pain [14]–[19]. In particular, a morphometric study by Granziera et al. found structural abnormalities in the visual network of motion-processing areas in patients with migraine, providing a noninvasively acquirable migraine biomarker for researchers [20]; DaSilva et al. showed that migraine sufferers had a thicker somatosensory cortex [21]. Here, we hypothesized that the brain cortical networks would progressively reorganize in individuals with migraine as the result of long-term and high-frequency headache attacks. Valfrè et al. (2008) observed that gray matter abnormalities were associated with the duration of migraine, indicating that migraine is a progressive disease and gets worse over time [3]. Progression of this disease could be depicted by alterations in the topological properties of the brain's networks [22]. It would be very important to investigate how the brain functional and structural network organization is altered by frequent cortical overstimulation associated with headaches.

To be able to fully understand structural/functional connectivity patterns, a comprehensive map of connection patterns of the human brain is needed [23]–[25]. The recent application of graph theory analysis (GTA), which defines a graph as a set of nodes (brain regions) and edges (functional connections), has become a powerful tool to investigate complex brain networks on a whole brain scale [23], [26]. While a graph serves as a powerful representation for characterizing the topological properties of brain networks [24], [25], it describes the basis of cognitive processing for distributed functional interactions between brain regions [27]. Structural connectivity networks are constructed from the measure of morphological association [28], and functional connectivity networks are based on correlations between functional MRI signals from different brain regions [29], [30]. Previously, our group considered gender-related differences in the topological property of resting networks in migraine sufferers and found widely distributed disorganization in their whole-brain networks. We found that migraine may have an additional influence on females and lead to more dysfunctional organization in their resting functional networks [29]. To test our hypothesis, we compared the intrinsic brain networks between female patients with migraine (PM) and gender-matched healthy controls (HC). Between-group differences and their associations with clinical variables were investigated.

## Materials and Methods

All research procedures were approved by the West China Hospital Subcommittee on Human Studies and were conducted in accordance with the Declaration of Helsinki. All participants in our study gave written informed consent.

### 2.1 Participants

Forty-three right-handed migraine patients (female, 32.6±11.1 years (mean age ± SD), 14.5±6.8 years (mean migraine duration± SD)) were recruited (Table 1). The migraine patients without aura fulfilled the ICHD-II criteria. Inclusion criteria for the PM group were according to Detsky et al. (2006): 1) It is a unilateral and/or pulsating headache; 2) Headache attacks last 4–72 hours (untreated or unsuccessfully treated); 3) There is nausea and/or vomiting, photophobia and phonophobia during headache and 4) Headache is disabling [31]. During the past 4 weeks, patients carefully rated the average pain intensity of the attacks (5.5±1.6, 0–10 scale, 10 being the most intense pain imaginable), migraine attack frequency (4.3±2.1 days/month) and migraine attack duration (13.7±9.9 hours). All patients had been free from a typical migraine attack for at least 1 week prior to MRI examination. Forty-three age-, education- and gender-matched, healthy, right-handed controls (age 33.4±10.2 years) were recruited from the local community. The controls neither had any headache days per year nor had family members who suffered regularly from a migraine or other headaches.

**Table 1:** Demographic characteristics of subjects.

Information	Healthy controls (n = 43 )	Patients with migraine (n = 43)	*p*-value
Age (years)	33.4±10.2	32.6±11.1	0.4
Education (years)	11.9±6.3	12.2±7.4	0.4
Disease duration (years)	N/A	14.5±6.8	-
**Migraine attacks during past four weeks**			
Attack duration (hours)	N/A	13.7±9.9	-
Attack frequency (times)	N/A	4.3±2.1	-
Average pain intensity (0–10)	N/A	5.5±1.6	-

For both groups, exclusion criteria were: 1) macroscopic brain T2-visible lesions on MRI scans; 2) existence of a neurological disease; 3) pregnancy or menstrual period; 4) use of prescription medications within the last month; 5) alcohol, nicotine or drug abuse; and 6) claustrophobia. All subjects gave written, informed consent after the experimental procedures had been fully explained.

### 2.2 Data Acquisition

All subjects underwent a resting-state functional MRI scan using a 3T magnetic resonance system (GE EXCITE, Milwaukee, Wisconsin) with an 8-channel phased array head coil. Prior to the functional run, a high-resolution structural image for each subject was acquired using three-dimensional MRI sequences using an axial Fast Spoiled Gradient Recalled sequence (3D-FSGPR) (matrix 256×256; FOV  = 256 mm ×256 mm; spatial resolution = 1 mm ×1 mm ×1 mm; TE  = 7.8 ms; TR = 3.0 ms). The functional images were obtained with an EPI (30 continuous slices with a slice thickness = 5 mm, TR = 2,000 ms, TE = 30 ms, FA  = 90°, FOV  = 240×240 mm^2^, data matrix = 64×64). For each subject, a total of 205 volumes were acquired, resulting in a total scan time of 410 s. Subjects were instructed to rest with their eyes closed, not to think about anything in particular, and not to fall asleep. After the scan, the subjects were asked whether they remained awake during the whole procedure.

### 2.3 Measure of gray matter volume

The structural images were processed using voxel-based morphometry (VBM) with Statistical Parametric Mapping-5 (SPM5) (http://www.fil.ion.ucl.ac.uk/spm). The average gray matter volumes were estimated. Specifically, the structural images were first corrected for non-uniformity artifacts; second, by using nonlinear normalization, the corrected images were registered to an asymmetrical T1-weighted template; third, the gray matter, white matter, and cerebrospinal fluid were obtained from then normalized images; and fourth, the resulting gray matter images were smoothed by a 4 mm isotropic Gaussian kernel.

### 2.4 fMRI data preprocessing

Image preprocessing was carried out using SPM5. The first five volumes were discarded to eliminate non-equilibrium effects of magnetization and allow subjects to get used to the scanning environment. Data preprocessing procedures included slice timing, realignment, and normalization: first, all datasets were initially corrected for temporal offsets using sinc interpolation and head movement-related effects using a six-parameter spatial transformation [32]; second, to minimize movement artifacts, individuals with an estimated maximum displacement in any direction larger than 1.5 mm or head rotation larger than 1.5° were discarded from the study. No data were excluded under this criterion; third, all datasets were spatially normalized to the Montreal Neurological Institute (MNI) echoplanar imaging template image and resampled to 2-mm isotropic voxels; and finally, a band-pass filter (0.01 Hz <f<0.1 Hz) was applied to remove the effects of low-frequency drift and high frequency physiological noise.

### 2.5 Defining nodes for cortical network analysis

In the network analysis, nodes must be first defined, and in this case, automated anatomically labeled (AAL) template images were used to parcellate the entire cerebral cortex into 90 anatomical regions of interests (ROIs) (Table 2)[33]. These 90 brain regions were considered as a set of nodes in our network analysis. This definition mode is widely used in current network studies [29], [30], [34].

**Table 2:** Cortical and subcortical regions defined by the AAL template image in standard stereotaxic space.

Region	Abbreviation	Region	Abbreviation
Superior frontal gyrus, dorsolateral	SFGdor	Superior temporal gyrus	STG
Superior frontal gyrus, orbital	ORBsup	Superior temporal gyrus, temporal pole	TPOsup
Superior frontal gyrus, medial	SFGmed	Middle temporal gyrus	MTG
Superior frontal gyrus, medial orbital	ORBsupmed	Middle temporal gyrus, temporal pole	TPOmid
Middle frontal gyrus	MFG	Inferior temporal gyrus	ITG
Middle frontal gyrus, orbital	ORBmid	Heschl gyrus	HES
Inferior frontal gyrus, opercular	IFGoperc	Hippocampus	HIP
Inferior frontal gyrus, triangular	IFGtriang	Parahippocampal gyrus	PHG
Inferior frontal gyrus, orbital	ORBinf	Amygdala	AMYG
Gyrus rectus	REC	Insula	ANG
Anterior cingulate gyrus	ACG	Thalamus	THA
Olfactory cortex	OLF	Caudate nucleus	CAU
Superior parietal gyrus	SPL	Lenticular nucleus, putamen	PUT
Paracentral lobule	PCL	Lenticular nucleus, pallidum	PAL
Postcentral gyrus	PoCG	Calcarine fissure and surrounding cortex	CAL
Inferior parietal gyrus	IPL	Cuneus	CUN
Supramarginal gyrus	SMG	Lingual gyrus	LING
Angular gyrus	ANG	Superior occipital gyrus	SOG
Precuneus	PCUN	Middle occipital gyrus	MOG
Posterior cingulate gyrus	PCG	Inferior occipital gyrus	IOG
Precentral gyrus	PreCG	Fusiform gyrus	FFG
Supplementary motor area	SMA	Rolandic operculum	ROL
Median- and para-cingulate gyrus	MCG		

### 2.6 Defining edges for cortical network analysis

For brain structural networks, the average gray matter volumes within each ROI (n = 90) were calculated. We measured the correlations based on the ROIs' averaged gray matter volumes [28], [35]. For each group, we obtained a structural connection matrix (90×90) across individuals between all possible connections of the node pairs.

For brain functional networks, we measured the regions' mutual association, expressing their functional coupling to define the network connection [25]. The mean time courses from deep white matter, ventricles and the 6 rigid-body motion parameters were regressed from the fMRI time series of 90 ROIs. After that, we computed the mean time series of each seed region and obtained a 90*90 matrix of the Pearson correlation coefficients between all possible connections of node pairs. The correlation coefficient was preserved as the functional connective intensity between the two regions.

The structural/functional connections of the cortical networks were defined if the correlation coefficient in the above structural/functional connection matrix between the two nodes achieved a correlation threshold. In the current study, the sparsity value used a network threshold which was defined as the total number of edges in a network divided by the maximum possible number of edges [36], resulting in cortical networks that had the same number of connections. Thus, it made cortical networks in the HC and PM groups to have the same network wiring cost [35]. We thresholded each correlation matrix repeatedly over a wide range of sparsity values ranging from 0.15 to 0.3 in 0.01 increments.

### 2.7 Network properties

The clustering coefficient of a node 0< Ci <1 is a ratio that defines the proportion of possible connections that actually exist between the nearest neighbors of a node [37]:
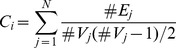



where N is the total number of nodes in the network, 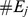 is the number of edges connecting the neighbors of node j, and 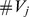 is the number of neighbors of node j.

The mean clustering coefficient of network C is the average over each node's clustering coefficient:
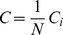



The minimum path length 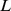 is the average of the shortest path lengths over each possible pair of vertices [37]:
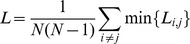



where 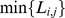 is the shortest path length between the 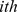 node and the 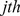 node, and the path length is defined as the number of edges included in the path.

Corresponding parameters for a random graph of C and L with the same number of nodes were also calculated, as denoted by 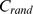 and 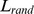. A graph is considered small-world if its average clustering coefficient C is significantly higher than a random graph constructed on the same number of nodes, and if the graph has a small average shortest path length. We examined the ratio 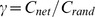 and the ratio 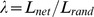 in our resting networks. In a small-world network, we expected the ratio to be 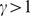 and the ratio 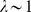 [37], [38].

### 2.8 Nodal centrality

The betweenness centrality of the brain regions was defined as the number of shortest paths between any two nodes in the network that pass through that particular node [39], which could assess the degree of information flow of a brain region in the cortical networks:
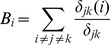



where 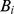 is the betweenness centrality of a node i, 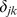 is the shortest path number from node *j* to node *k*, and 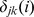 is the shortest path number from node *j* to node *k* that passes through node *i*.

### 2.9 Network modularity

Modularity Q of the brain cortical networks is defined as 
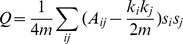
, where 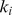 and 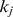 are the degrees of nodes *i* and *j*, 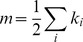 is the total number of edges in the network, and 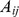 is the number of edges between the 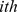 node and 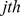 node. If the network could be divided into two groups, 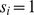 indicates that node *i* belongs to group 1 and 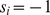 indicates that node *i* belongs to group 2. The expected number of edges linked at random between nodes *i* and *j* is 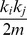 [40], and the modularity Q quantifies the difference between the number of edges within the actual module and those that are randomly connected. Therefore, the module evaluation is to find the optimal partition that could result in the largest network modularity [41]. The modularity matrix resulting from the algorithm is related to the modularity score. There were 1000 ranked solutions from the connection matrices; the optimal solution was chosen.

### 2.10 Statistical analysis

**Figure 1:**
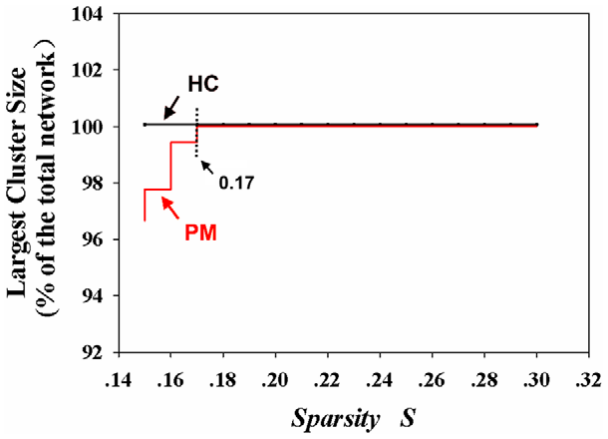
Largest cluster size as a function of sparsity S for the healthy controls' (HC) structural networks (black line) and patients' with migraine (PM) structural networks (red line). S = 0.17 is the lowest sparsity value that could guarantee each network was fully connected with all of the nodes.

To estimate whether there existed significant group differences in the functional connections, a recently developed network-based statistic (NBS) was used in the current study [42]. To evaluate whether there existed significant group differences in the structural/functional network properties (the mean clustering coefficient C, the mean minimum path length and degree centrality), nonparametric permutation tests were used in the current study [43]. The false discovery rate was used to correct the multiple comparisons. A fixed sparsity value S = 0.17 was selected as being typical in the network analysis (Fig. 1).

## Results

### 3.1 Abnormal topological properties in cortical networks

**Figure 2:**
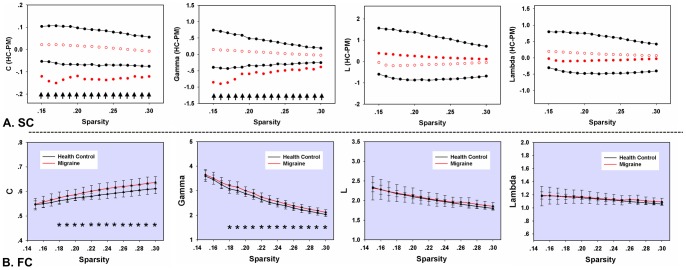
Between-group differences in the mean clustering coefficient C, normalized clustering coefficient gamma, the shortest path length L, and normalized shortest path length lambda over a range of sparsity values. (A) Differences between the HC and PM groups in subjects' structural networks. The black solid points represent the 99% confidence intervals of the between-group differences obtained from 5000 permutation tests at each sparsity value. The red open circles describe the mean values and the red solid points indicate significant between-group differences in network metrics. (B) Differences between the HC and PM groups in the subjects' functional networks. The red lines represent the network metrics in the PM. The black lines describe the network metrics in the HC. The horizontal stars indicate the significant between-group differences (*p*<0.01, FDR corrected).

**Figure 3:**
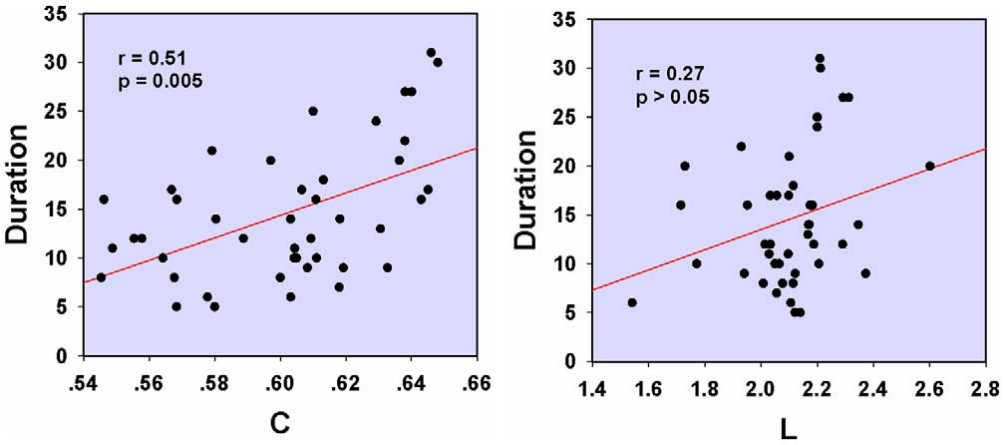
Correlation between the mean clustering coefficient C/the shortest path length L and migraine duration while controlling for patients ' **age.** Significant correlation was found in the mean clustering coefficient (r = 0.51, *p* = 0.005), but not in the shortest absolute path length L.

In our results, we constructed the structural/functional connectivity networks at sparsity values ranging from 0.15 to 0.3, and the small-world properties were obtained at different thresholds respectively (Fig. 2). The typical features of small-world properties were found in both structural and functional connectivity networks. Compared with random networks, cortical networks had higher mean clustering coefficients (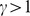), but with a similar shortest absolute path length (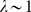). As shown in Figure 2A, the mean value of the clustering coefficient C (*p*<0.01, corrected) and normalized clustering coefficient 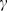 (*p*<0.01, corrected) were significantly higher in PM structural networks on a whole sparsity range. No significant differences were found in the shortest absolute path length L and 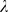 (*p*>0.05). Moreover, similar results were found in patients' functional networks. The comparisons revealed significantly increased clustering coefficients C and 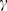 (*p*<0.01, corrected), but they had an unaltered shortest absolute path length as compared with HC. Furthermore, the mean clustering coefficient C was positively correlated with the duration of migraine in the patients' functional networks (Figure 3, *r* = 0.51, *p* = 0.005, controlling for age). No significant correlation was found in the shortest absolute path length L (Fig. 3).

### 3.3 Abnormal nodal centrality

**Figure 4:**
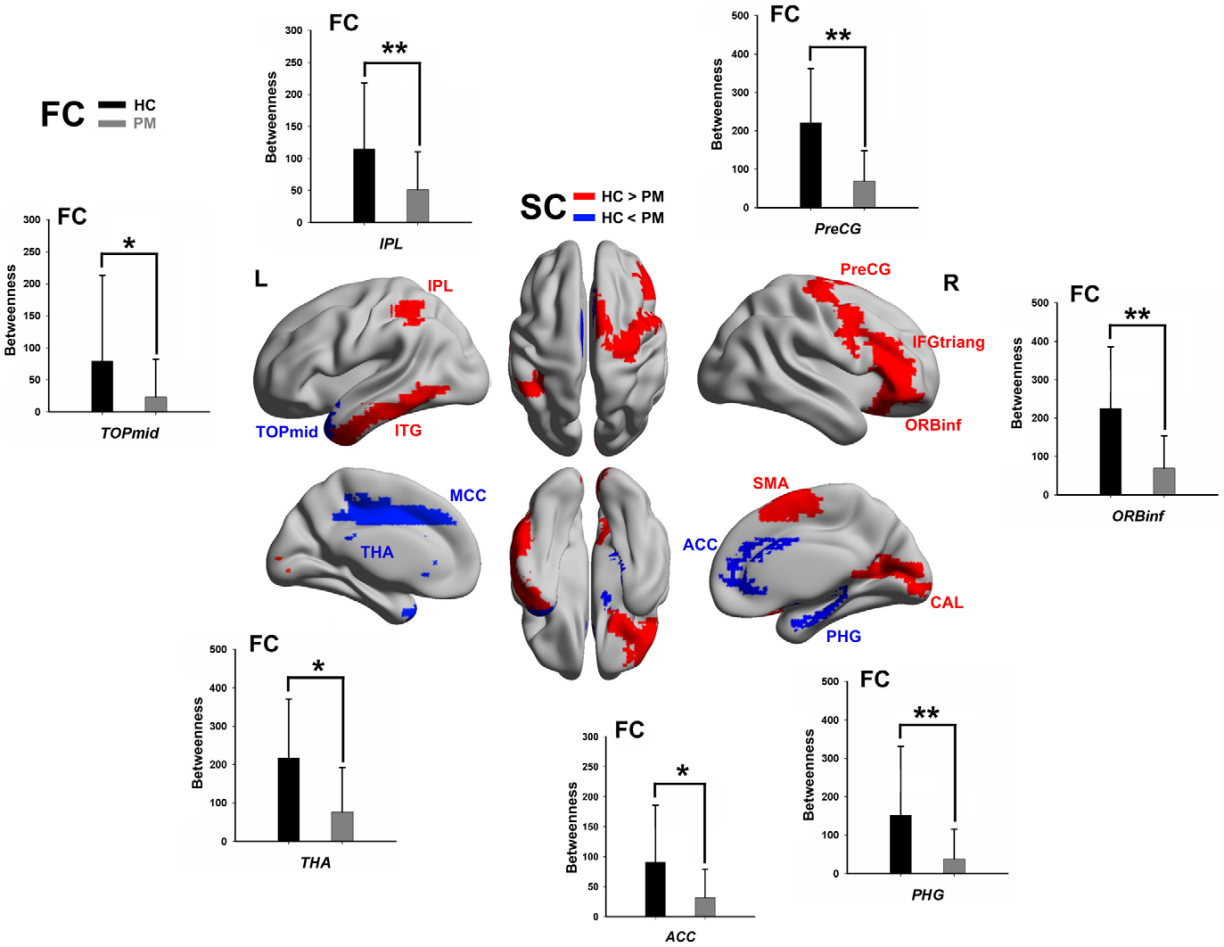
Significant between-group differences of betweenness centrality. Regions with abnormal betweenness centralities in patients' structural networks were rendered on the brain surface by visualizing it with the BrainNet viewer (red, HC>PM; blue, HC< PM). The black and grey bar graphs indicate dysregulated brain regions in patients' functional networks (nonparametric permutation test, *p*<0.01, corrected).

Twelve regions exhibited a betweenness abnormality in PM structural networks (*p*<0.01, corrected, Fig. 4), including the inferior parietal gyrus (IPL), inferior temporal gyrus (ITG), temporal pole of the middle temporal gyrus (TPOmid), precentral gyrus (PreCG), triangular part of the inferior frontal gyrus (IFGtriand), orbital part of the inferior frontal gyrus (ORBinf), supplementary motor area (SMA), calcarine (CAL), anterior cingulate gyrus (ACG), parahippocampal gyrus (PHG), median- and para-cingulate gyri (MCG) and thalamus (THA); eight regions revealed significantly decreased betweenness centrality in PM functional networks (*p*<0.01, corrected), including the PreCG, dorsolateral part of the superior frontal gyrus (SFGdor), PHG, ACG, THA, amygdala (AMYG), TPOmid and IPL. Furthermore, some of these regions showed a significant correlation with migraine duration (Table 3). We found that seven regions commonly had abnormal betweenness centrality across the structural and functional connectivity networks (PreCG, ORBinf, PHG, ACG, THA, TPOmid and IPL).

**Table 3:** Foci with significant changes in the betweeness centrality from normal controls (NC) versus patients with migraine (PM).

Regions	*NC(FC)*	*PM(FC)*	*corrected p (NC vs. PM)*	*correlation with migraine duration*	
	mean ± sd	mean ± sd	value	r	p
PreCG_r	221.2±138.8	69.1±79.2	<0.05	−**0.48**	**0.002**
ORBinf_r	225.5±160.2	69.5±84.2	<0.05	−**0.5**	**0.001**
SFGdor_l	252.2±124.8	107.5±115.8	<0.05	−0.11	>0.05
PHG_r	151.8±179.3	38±77.2	<0.05	−0.1	>0.05
ACG_r	90.7±95	31.8±47.1	<0.05	−**0.34**	**0.03**
THA_r	217.9±153.6	76.8±115	<0.05	−**0.35**	**0.03**
AMYG_r	42±65.5	10±23.7	<0.05	−**0.33**	**0.04**
TPOmid_l	79.4±134	23.1±59	<0.05	−0.07	>s0.05
IPL_l	114.5±103.3	51.4±59.3	<0.05	−0.24	>0.05

### 3.4 Dysregulated interregional correlations

**Figure 5:**
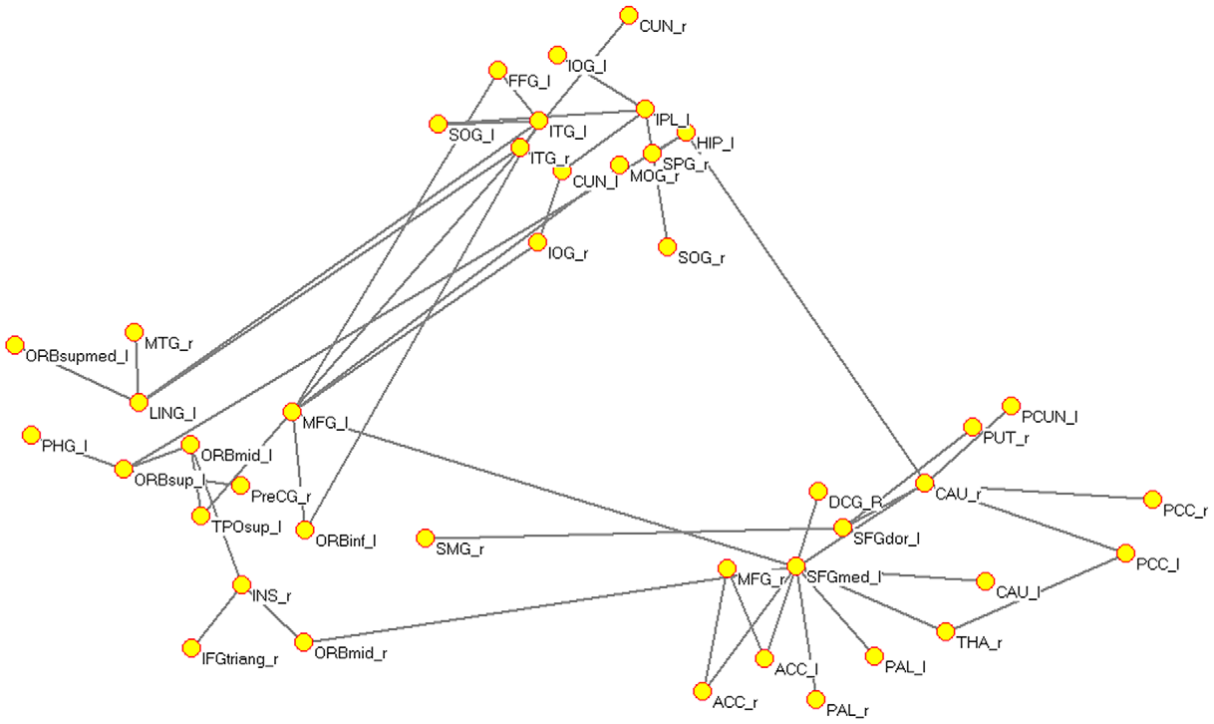
Significant between-group differences in the intensity of the brain connections in PM structural networks (nonparametric permutation test, *p* = 0.005). For the abbreviations of the regions, see Table 2.

In the PM structural networks, several pairs of connections were significantly altered (nonparametric permutation test, *p* = 0.005). We focused on the largest connected component, and these abnormal connections were widely distributed in the patients' structural networks including connections between different lobes, mainly in the ACC, THA, hippocampus (HIP), caudate (CAU), putamen (PUT), pallidum (PAL), orbital part of the prefrontal cortex, parietal lobule, temporal lobes, and occipital cortex. All of these connections revealed increased values in patients compared with HC (Fig. 5).

**Figure 6:**
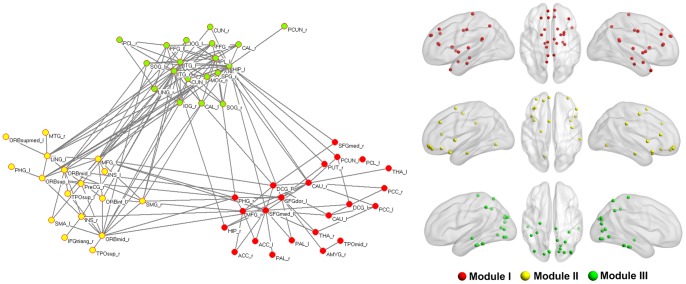
Significant between-group differences in the intensity of the brain connections in PM functional networks (NBS, *p* = 0.005). These dysfunctional connections in patients' cortical networks were formed into a connected component and three dysregulated communities were identified. Besides the topological space, the brain regions were also projected onto the brain surface according to their MNI centroid stereotaxic coordinates. Different colors represent distinct modules. For the abbreviations of the regions, see Table 2. This figure was visualized with the BrainNet viewer.

**Figure 7:**
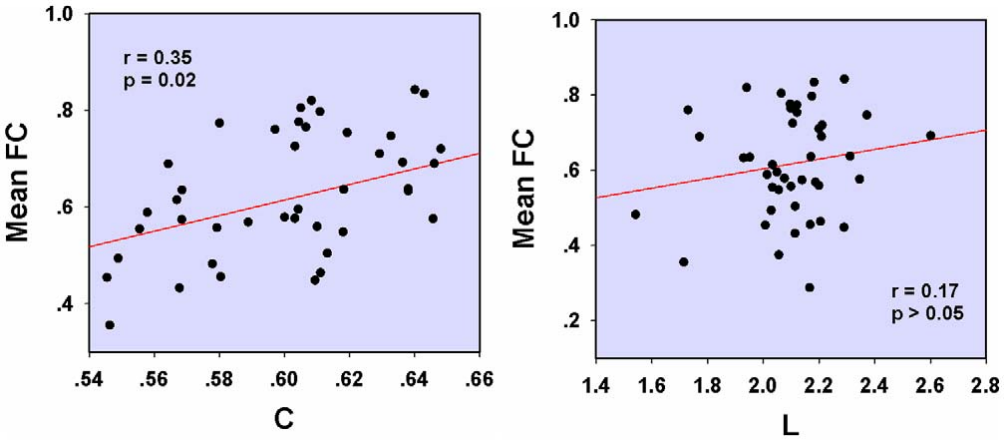
Correlation between the mean clustering coefficient C/the shortest path length L and the mean connectivity values of dysfunctional connections in the patients ' **functional networks.**

In the PM functional networks, a single component network was found to be most significantly altered in the PM by using NBS (*p* = 0.005). The functional network of patients was more dysregulated as compared with their structural networks, and connections showed significant increases in interregional correlations (Fig. 6). Furthermore, mean connectivity values of these dysfunctional connections were significantly correlated with the mean clustering coefficient C of their networks (*r* = 0.35, *p*<0.05) (Fig. 7).

### 3.5 Modularity

Modularity is defined as a set of nodes the have many intra-modular connections but sparser inter-modular connections, indicating a decomposability of the system into smaller subsystems [23]. As shown in our results, optimal modularity was achieved in three modules (Fig. 6). Module I included 23 regions (red), including the prefrontal cortex, ACC, MCG, PCG, PHG, HIP, AMYG, thalamus, pallidum, caudate and putamen, which were largely considered to be involved in pain processing [44]. Module II (yellow) showed a cluster mainly in the orbital part of the prefrontal cortex, insula, temporal cortex and sensorimotor cerebral cortex. Module III (green) exhibited brain regions that predominantly consisted of the occipital gyrus, cuneus, lingual and inferior temporal gyrus, which are areas recognized as the visual cortex.

## Discussion

In this study, we presented graph theoretical analyses of topological properties of whole-brain networks by examining the structural and functional connectivity network disorganization in migraine sufferers as compared with HC. Exploring the similarity of functional and structural network changes, we found that migraine disrupts the topological organization of cortical networks, and the vital brain hubs related to pain-processing revealed abnormal nodal centrality in both structural and functional networks; brain alteration occurring in patients with migraine was not limited to the local abnormal CNS but to a disruption in the topological organization of intrinsic whole-brain networks. Furthermore, disrupted information exchange between brain areas was reorganized into a hierarchical modular structure. Taken together, our results indicated that long-term and high-frequency headache attacks may lead to pathological cortical network reorganization in PM, reflecting abnormal brain dynamics due to the effects of brain disease. It has profound implications for our understanding of topological organization of complex brain networks in migraine.

In our findings, both structural and functional connectivity networks of the PM showed significantly increased clustering coefficients over a wide range of sparsity values as compared with HC. Given that the clustering coefficient is an index of local structure, such results could be attributed to the increased degree that more brain regions in patients tend to cluster together, involving networks comprised of pain processing and the visual cortex (Fig. 5 and Fig. 6). These topological features in migraine-affected brain networks potentially indicated substantial reorganization of cortical networks in a long-term pathological condition. Several studies demonstrated that the brain region within the structural core was composed of a number of highly connected and highly central neocortical hub regions that linked all of the major structural modules, thus playing an important role for information flow between separate groups of brain regions [10], [45]. Nodal centrality, a powerful measure for the relative importance of a node in a network, has been applied to evaluate the information integration ability of a brain region in patients' cortical networks. Compared with the HC group, nine regions exhibited abnormal centrality in both structural and functional connectivity networks in PM (the PreCG, ORBinf, PHG, ACG, thalamus, TPOmid and IPL) (Fig. 4 and Table 3). Moreover, we found that betweenness values were negatively correlated with the duration of disease in the PreCG, thalamus, ACG and ORBinf. Patients with migraine exhibited a focal gray matter decrease in the ACG and abnormal activation during task-related functional MRI or in a resting state together with other pain-related areas (PreCG and thalamus) [3], [5], [17]–[19]. A recent fMRI study reported that migraine patients had a significant decrease in regional homogeneity values in the orbital part of the prefrontal cortex [5]. On the other hand, Kaiser et al. suggested that the generation of cortical hubs was governed by the development of the human brain to achieve optimal information transmission [46].Our results are compatible with these previous findings. Decreased nodal centralities of these regions in PM cortical networks indicated their structural and functional dysfunctions in integrating diverse global information in the pain-processing network.

In our results, compared with the matching HC, several abnormal interregional correlations were found in PM cortical networks and were formed into a large connected network, which refers to several brain circuits (Fig. 5 and Fig. 6). Notably, abnormal connectivities in the chronically migraine-inflicted brain have a skewed balance between structural and functional networks. More dysfunctional connections were found in the patients' functional brain networks (Fig. 6). Our findings revealed migraine affected brain topology and regional interaction in both structural and functional connectivity networks. Computational work suggested that the underlying structural connectivity networks may shape functional connectivity networks on multiple time scales [8], [47]. Recently, several studies also found that the coupling of functional and structural connectivity networks was significantly disrupted in disease-specific states [12], [48]. Park et al. suggested that anatomical connectivity, as a major constraint of functional connectivity, has a relatively stable structure; the functional connectivity network is relatively flexible [49]. According to our results, migraine affected structural and functional brain networks acting differently in the organizational patterns of brain cortical networks in PM. Functional connectivity changes were more complex. We inferred that the structural connectivity networks may be less affected in PM; however, the functional connectivity networks may be more sensitive to long-term headache attacks and therefore suffer more abnormal brain connections.

Prior studies have noted the importance of modular structure in the biological networks which may arise from natural selection pressure or evolutionary constraint for adaptation to environmental demands [41], [50]–[52]. From clinically-based studies, subclinical posterior circulation strokes and diffuse white matter lesion load increased with the frequency of migraine [53], which suggested that migraine may lead to progressive alteration in the brain [54]. In our study, we found that modular structures existed within the dysregulated brain networks in PM (Fig. 6). Three dysregulated communities were identified in the connected component in PM that confer with several subsystems mentioned in earlier studies in headaches (Fig. 6), such as in the pain-related information processing and motion-processing visual network [1], [13]. These results reflect a fundamental principle of migraine-related alteration in the balance of functional segregation and integration for the long period of experiencing and anticipating a headache, and exhibiting the consequences of the secondary effect of having migraine. He et al. (2009) reported that there was highly organized modular architecture in the functional networks of the healthy human brain, and they speculated that such module-specific brain organization may be due to an evolutionary conserved pattern in human functional brain maturity over development [41]. According to our findings, the formation of a dysregulated cortical network may be constrained by migraine-influenced network dynamics, and such pathological network dynamics may progressively reshape the network organization into a hierarchical modular structure. While brain injury becomes progressively worse, it may be accompanied by light to heavy and infrequent to frequent headaches and other clinical symptoms in the patients' daily lives [54]. Our results provide new insight into migraine-related brain network reorganization and support the concept that migraine may be a progressive disorder.

There are several issues that need to be further addressed in the present study. First, in our network analysis, we employed AAL template images and had 90 ROIs covering the entire cerebral cortex which were used in several published studies [41], [55]. However, different parcellation strategies of graph analytical techniques may result in distinct topological architecture [56]–[59]. To test the reproducibility of our results, future studies need to consider the effect that a specific parcellation approach has on graph analytical findings. Secondly, aside from a chronic headache attack, one major differentiating feature between healthy controls and migraine patients was the use of powerful drugs for treating migraine. These drugs have additional effects on brain structural and functional connectivity. Thus, this may also be a confound in our study. To test the reproducibility of our results, longitudinal studies are needed. Thirdly, migraine is a predominantly genetically determined disorder, and a recent study found that a flawed gene in a family of migraine sufferers could trigger severe headaches. Hence, whole-brain network dysregulation should be considered in a migraine-related genetic risk. There is the high possibility that migraine may have a different influence on the organization of cortical networks for individual genetic variants.

In summary, by exploring the topological properties of the structural and functional connectivity networks in patients with migraine, the patients showed a dysregulated brain organization in their cortical networks. This migraine-affected brain alteration had a skewed balance between structural and functional connectivity networks in patients. More abnormal brain connections were found in migraine sufferers; furthermore, disrupted information exchange between brain regions was formed into a hierarchical modular structure. Our results provide valuable insights into the understanding of brain network reorganization that could be attributed to developmental aberration and the underlying pathophysiology resulting from migraine. In our future longitudinal study, direct evidence will be provided to prove that migraine is a progressive disease and results in dysregulated brain network reorganization.
